# Visualisation of BioPAX Networks using BioLayout *Express* ^3D^

**DOI:** 10.12688/f1000research.5499.1

**Published:** 2014-10-20

**Authors:** Derek W. Wright, Tim Angus, Anton J. Enright, Tom C. Freeman

**Affiliations:** 1 The Roslin Institute and Royal (Dick) School of Veterinary Studies, The University of Edinburgh, Midlothian, Scotland, EH25 9RG, UK; 2 EMBL-European Bioinformatics Institute, Wellcome Trust Genome Campus, Hinxton, Cambridge, CB10 1SD, UK

**Keywords:** signalling network, analysis, proteins, bioinformatics

## Abstract

BioLayout *Express* ^3D^ is a network analysis tool designed for the visualisation and analysis of graphs derived from biological data. It has proved to be powerful in the analysis of gene expression data, biological pathways and in a range of other applications. In version 3.2 of the tool we have introduced the ability to import, merge and display pathways and protein interaction networks available in the BioPAX Level 3 standard exchange format. A graphical interface allows users to search for pathways or interaction data stored in the Pathway Commons database. Queries using either gene/protein or pathway names are made via the cPath2 client and users can also define the source and/or species of information that they wish to examine. Data matching a query are listed and individual records may be viewed in isolation or merged using an ‘Advanced’ query tab. A visualisation scheme has been defined by mapping BioPAX entity types to a range of glyphs. Graphs of these data can be viewed and explored within BioLayout as 2D or 3D graph layouts, where they can be edited and/or exported for visualisation and editing within other tools.

## Introduction

There has been an explosion in the amount of publicly available pathway and interaction data in recent years, derived from high-throughput experimental techniques, such as two-hybrid systems, mass spectrometry, phage display etc., or through focused studies and manually curated from the literature into pathway models ^1– 3^. There are many resources that store such data: at the time of writing, the website pathguide.org ^4^ listed 547 pathway and interaction databases. However, many of these resources store the data in idiosyncratic formats and as a result it has been difficult for resources to exchange data between them.

To address this problem, there have been a number of efforts to standardize the exchange of pathway and protein interaction data from disparate sources, including PSICQUIC ^5^, CellML ^6^ and BioPAX ^7^. Of these, BioPAX is one of the most widely adopted data exchange formats. BioPAX is a community standard ontology for describing pathway and protein interaction data, suitable for qualitatively representing the current knowledge of biological systems. Seventy-four of the resources listed by the PathGuide currently support BioPAX, including some of the most widely used resources. BioPAX is overseen by the Computational Modeling in Biology Network (COMBINE) ( http://co.mbine.org/) and has been released in major versions referred to as levels. The latest release is BioPAX level 3, version 1.0.

Pathway Commons ^8^ is a publicly available resource that aggregates and integrates pathway data from multiple organisms and databases into a common BioPAX language linked data representation. Data stored within this resource are currently derived from the databases ChEBI ^9^, UniProt ^10^, Reactome ^1^, Pathway Interaction Database ^11^, PhosphoSite ^10^, HumanCyc ^12^, HPRD ^13^ and PANTHER ^2^. The Pathway Commons website provides query and bulk download of these data. The system also makes these data available via a REST web service API, which provides programmatic access to data over the web. Pathway Commons has recently been upgraded, supporting BioPAX Level 3 and providing advanced graph queries via the CPath2 REST API.

A range of software tools already supports BioPAX use and exchange. For example the network analysis tool Cytoscape ^14^ has support via plugins (known as “apps” in Cytoscape version 3) ^15, 16^. The BiNoM plugin ^17^ can import BioPAX Level 3 OWL files, the CyPath2 plugin is able to import and visualise BioPAX from the Pathway Commons resource, and the ChiBE pathway editor ^18^ allows users to visually edit BioPAX pathways. CellDesigner, a graphical pathway editor, can export pathways as BioPAX ^19^ and users of the R statistical programming language can access BioPAX via the rBiopaxParser package ^20^. As BioPAX is a language based on the semantic RDF/OWL standard, it can also be edited using standard ontology authoring tools such as Protégé and WebProtégé ^21^. However, it should be noted that there are various compatibility issues with some of the above, with different apps/tools being specific for different versions of the tools or BioPAX.

There is considerable interest in BioPAX data from the bioinformatics community and a growing interest in tools that support its visualisation and analysis. Here we report the implementation of a simple-to-use graphical interface within the network analysis tool BioLayout *Express* ^3D^ ^22, 23^ that now supports querying of the Pathway Commons resource, allowing the user to pull in the results of specific gene/protein- or pathway-centric queries, and to visualise the results in a graphically intuitive manner.

## Implementation

BioLayout *Express* ^3D^ version 3.2 has been developed to open BioPAX Level 3 OWL files and generate network graph visualisations of BioPAX encoded pathway or protein interaction data. A web service client has also been developed within BioLayout to query Pathway Commons and import BioPAX networks directly.

PaxTools ^24^, an open-source Java library for developing BioPAX applications, has been incorporated into BioLayout. When a BioPAX OWL file is opened, it is parsed using PaxTools and an in-memory object model containing the elements in the BioPAX document is created. If the BioPAX major version is lower than Level 3, the object model is upgraded to Level 3 before the graph is constructed. The program iterates through each entity, looking up and assigning a shape for that entity type then creating a corresponding graph node. The program then connects nodes by creation of edges to represent components that are members of a complex, steps of a pathway and participants of an interaction.

## Input options

**Figure 1.**
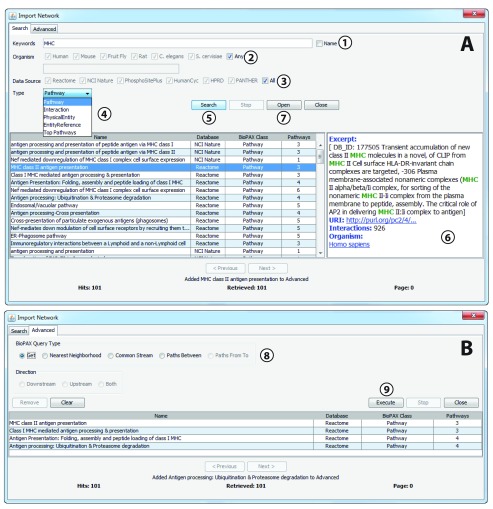
*Import Network* user interfaces with main features highlighted. ( **A**) *Import Network* search dialog. This dialog supports the querying of the Pathway Commons database. **1**. Keywords are entered into the *Keywords* field. To restrict the search to the BioPAX pathway name only (as opposed to all text associated with pathway), leave the ‘Name’ checkbox ticked. Terms may be separated with Boolean operators AND/OR and a specific search field may be combined with a search term separated by a colon (:) and wildcards may be searched using an asterisk (*); **2.** NCBI organism ID number or species name may be entered in the text field or for convenience popular species may be searched for using the checkboxes provided; **3.** Information from specific databases from which Pathway Commons aggregates may be selected, restricting searches to information provided by those resources; **4.** Dropdown list defines which BioPAX type to search for: *Pathway*, *Interaction*, *Physical Entity*, *Entity Reference*. There is an additional option, *Top Pathways*, which is a special case; this is defined as “pathways that are neither ‘controlled’ nor ‘pathwayComponent’ of another process”; **5.** Click the *Search* button to perform the search. Search results are displayed in the table. Results are returned in pages of 500 search hits; the sequence of pages may be navigated using the *Next*/ *Previous* buttons; **6.** Click on a row in the results table to display detailed information about that network in the pane on the right hand side; **7.** Click the *Open* button to download and display the network for the search hit you have highlighted in the results table; **8.** If you wish to perform an advanced graph query, double-click the row(s) in the results table and the search hits will be added to the *Advanced* tab. The *Advanced* tab of the *Import Network* dialog enables you to perform advanced graph queries on search merging networks etc.; **9.** When the procedure is defined, click *Execute* to visualise the results.

BioLayout *Express* ^3D^ communicates with Pathway Commons via the cPath2 REST API, sending commands using the PaxTools library. Using a search dialog ( Figure 1A), opened by selecting *“File -> Network From Public Database…”* within BioLayout, the user may refine searches by keywords, species, data source and BioPAX type. For convenience, predefined search options are provided for individual data sources and popular species. Queries may be for specific genes/proteins or for pathways, searching the entire record or just the title.

Search results are displayed in a table. Clicking on a search hit displays an excerpt from the description with the highlighted search term, the persistent Uniform Resource Identifier (URI) of the search hit on Pathway Commons, the number of interactions (for pathways only) and the species name, which are displayed in a panel alongside the table of hits. As the BioPAX document defines species using Identifiers.org ^25^ standard URIs, the scientific names of the species in the search results are looked up in the NCBI Taxonomy database ^26^ using the NCBI EFetch SOAP ^27^ web service. This live lookup ensures that BioLayout is capable of displaying the name of any species that may be found in the search results. For a pathway search hit, the number of interactions is calculated and displayed, so as to give the user an indication of the size of the network that will be produced (some hits may be very small and possibly not worth displaying). This value is obtained by counting the results of a traverse query of interactions within the selected pathway.

The user may choose to display the network for a single search hit, in which case BioLayout downloads the corresponding OWL file from Pathway Commons and opens it, displaying the network. Alternatively, a user may choose to select a number of search hits and then perform advanced graph queries on multiple hits, using the operations provided by the Pathway Commons cPath2 web service. A search hit may be added to the *Advanced* tab by double-clicking the row ( Figure 1B). Advanced query options are:

- *Get* – multiple networks combined into a single network
- *Nearest Neighborhood* – first order neighborhood of nodes within search hits
- *Common Stream* – common upstream or downstream of search hits
- *Paths Between* – network forming paths between search hits
- *Paths From To* – network forming the paths from search hits in rows selected by the user in the *Advanced* table to unselected search hits

## Visualisation of BioPAX data

A visual scheme has been defined, where node shapes and colours have been mapped to BioPAX entity types. BioLayout already supports the import of mEPN (modified Edinburgh Pathway Notation) pathway models ^3, 28^ saved as GraphML files. When visualised, the concepts supported by this pathway notation system are translated into equivalent 2D or 3D shapes. We therefore chose equivalent glyphs for BioPAX entities and concepts where possible, in order to provide a consistent user experience.

**Figure 2.**
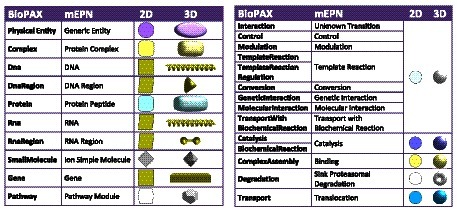
Table listing BioPAX concepts and the mapping on to the mEPN pathway notation scheme and their representation in BioLayout *Express* ^3D^ as 2D and 3D nodes.

Some BioPAX concepts could not be mapped to the existing mEPN scheme so new glyphs were added. For example, a dumbbell shape was added for *RNA-Region*. Some concepts did not have an exact analogue. In the case of the BioPAX *Small Molecule* type, the equivalent concept in mEPN could either be *Ion/Simple Molecule* or *Simple Biochemical*; the *Ion/Simple Molecule* glyph was used. The BioPAX ontology has a hierarchical structure with increasing levels of granularity. Some glyphs were added to mEPN to handle generic BioPAX types where the more detailed type is not available in the data, such as a *Control* transition. Mappings between BioPAX/mEPN concepts and the 2D and 3D shapes used to represent them are shown in Figure 2.

### Layout

**Figure 3.**
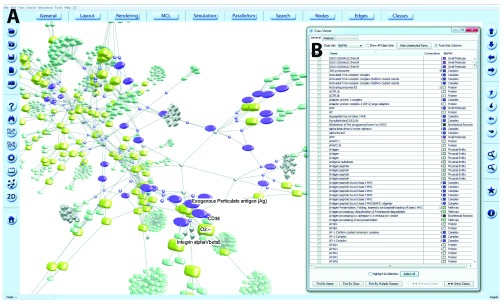
Visualisation of BioPAX networks in BioLayout *Express* ^3D^. ( **A**) 3D network view of a BioPAX query. Different entity/concept types defined in BioPAX Level 3 are translated into nodes of different size, shape and colour depending on type. Graphs may be explored, edited and names displayed. Options for these functionalities are contained within the BioLayout menus. ( **B**) The names and node types of selected nodes may be viewed with the *Class Viewer*, which also provides information on the number of edges. Within this window nodes may be selected/deselected, searched by name or class and information exported.

BioPAX may describe the interaction between the components of a pathway but it does not define layout co-ordinates for visualisations, even if the original source of the information, such as Reactome, contained this information. In the absence of layout information, a graph layout must be computed algorithmically. We recommend the Fast Multipole Multilevel Method (FMMM) layout algorithm, implemented within BioLayout, for use with BioPAX networks ^29^. FMMM is a force-directed layout algorithm, introduced in BioLayout version 3.1 that allows graph layout to be computed highly efficiently, with a small number of iterations. The algorithm produces elegantly laid out graphs ( Figure 3A) in both 2D and 3D, with sparsely arranged nodes and is particularly useful for the visualisation of large structured networks, such as those obtained from BioPAX.

## Exploration of networks

BioLayout’s *Class Viewer* ^23^ is used as an inspector for the graph. The *Class Viewer* enables a graph to be sub-categorized into classes, based on node type. The classes taken together form a *Class Set*. During the graph construction process, a Class Set is created for BioPAX features and as the graph nodes are created, each node is assigned to a class with the name of the BioPAX entity type to which it corresponds ( Figure 3B).

The *Import Network* search dialog may be opened from within the *Class Viewer*, while navigating a gene co-expression network, using the *Search Database* function. This opens the dialog with the *Keywords* field pre-populated with gene names from selected nodes within the graph, enabling the user to search for pathways that involve the genes of interest. This represents a means of directly integrating genomic data with pathway data. Similarly, an analysis of a gene expression network or similar, e.g. clustering of co-expression modules, may be exported ( *File -> Export -> Class Sets As File*) and then, assuming that the node identifiers are the same between the two networks, imported ( *File -> Import -> Class Sets…*) whilst visualising a BioPAX network. In this way, the genes of interest in the expression network can be highlighted on the interaction network or *vice versa*.

## Conclusion

There is now a wealth of pathway and protein interaction data in the public domain, collected and curated at great expense. However, accessing and using these data has proved challenging for many due to the lack of standard formats for data exchange between resources. The BioPAX standard has gone a long way to resolve this issue and has been widely adopted by the community. The Pathway Commons database has therefore been able to amalgamate the information stored in a number of the main pathway/interaction resources, making the information available through the CPath2 web service. Here we report our implementation of data query and import functionality within BioLayout *Express* ^3D^ version 3.2, thereby leveraging a powerful tool to support the visualisation and analysis of large pathway and protein interaction networks. The data stored in the Pathway Commons resource may now be easily searched and hits combined. The resulting networks can be displayed in 2D or 3D using a graphical display language that differentiates between the entity types described in the BioPAX hierarchy. Within the tool, the graphs can be explored and edited and where necessary exported for visualisation within other tools.

## Software availability

### Sofware access

Software and source code are available from http://www.biolayout.org.

### Archived source code as at the time of publication

http://dx.doi.org/10.5281/zenodo.12216 ^30^

### Software license

GNU Public Licence version 3 http://www.gnu.org/copyleft/gpl.html
