# Supplementary material for: Visualisation of BioPAX Networks using BioLayout Express 3D
Source: F1000Res. 2014 Oct 20;3:246. [Version 1] doi: 10.12688/f1000research.5499.1 (PMC4406191; doi:10.12688/f1000research.5499.1)
Supplement: Peer review history

# Peer review: Referee response for version 1

**DOI:** 10.5256/f1000research.5869.r8200

**Published:** 2015-04-07

**Authors:** Jianguo Xia

**Affiliations:** 1 Institute of Parasitology and Department of Animal Science, Faculty of Parasitology, McGill University, Montreal, QC, Canada

General Comments:

The BioLayout Express 3D is a very useful tool, especially its unique 3D rendering capability. The supports for importing network files in BioPAX level 3 format and integration with Pathway Commons appear to work well as described in the paper. My comments are mainly concerned with network visualization.

1. Navigation in large/dense networks: 
  Users often want to zoom in to a particular node/region of the network to view the connections and structures. It is difficult to achieve this using right-mouse button. A better approach may be to add support for three-wheel mouse – point the mouse to the node and then scroll to zoom in to the pointer direction.
2. Enhancing support for node search in a dense network:
  Users should be able to click to highlight AND zoom in to it.
3. Better scaling control (esp. for 2D view):
  Users would expect that node overlapping issue would be resolved by zooming in to the region. This is not the case during testing. Rather than simply getting bigger in size, both the sizes and relatives distance between nodes should be scaled properly when users zoom in. If there are some advanced options to achieve this effect, the default parameters should be improved.

 

Bugs (Version 3.3, Mac version)

On the 2D view, select and drag a node to a new position, the mouse pointer and the node are not well synchronized during the process.  Black screen also occurs sometimes. 

Additional comments / wish list:

1. In addition to comparing their abilities in supporting BioPAX format, a general discussion on network visualization features of these popular tools will be very useful for new users to make informed choices.
2. The standalone tools have the advantage for dealing with large data. The current tool can be improved with regard to more layout options and better performance for large networks.
3. Given the increasing popularity for web-based visualization, the support for exporting the graph for D3.js rendering is certainly a welcome step. An online version for 3D network visualization (maybe based on WebGL?) will be highly received by the community. 

I have read this submission. I believe that I have an appropriate level of expertise to confirm that it is of an acceptable scientific standard.

# Peer review: Referee response for version 1

**DOI:** 10.5256/f1000research.5869.r6455

**Published:** 2014-10-27

**Authors:** Gary Bader

**Affiliations:** 1 Donnelly Centre for Cellular and Biomolecular Research (CCBR), University of Toronto, Toronto, ON, M5S 3E1, Canada

The authors describe BioPAX loading and visualization support for the established BioLayout Express 3D network visualization software. As far as I know, this is the only software capable of 3D display of BioPAX derived networks. The new feature seems to work very well as described. I have a few minor comments to improve the manuscript.

Minor notes:

- “the persistent Uniform Resource Identifier (URI)” – URIs in Pathway Commons are not guaranteed to be consistent between releases. They can be persistent if they are externally standardized e.g. uniprot IDs, but internal pathway commons URIs may change from release to release.
- The manuscript would benefit from one biological use case. The highlighting expression data paragraph is interesting, but including a biological example would help potential biologist users know how to use the software for a particular analysis.
- It would be nice to see a future directions section e.g. is there a plan to visualize SIF networks derived from BioPAX?
- It would be useful to briefly mention some examples of information that is not covered in the BioPAX to mEPN mapping e.g. post-translational modifications on proteins, the directionality of edges e.g. that could distinguish between left and right participants of an interaction vs. all participants of an interaction. Some of this information is possible to show in other visualization options, such as the Systems Biology Graphical Notation, which the authors may want to reference.

 

Software comments:

- The top pathways feature requires entering a keyword before the Search button is active, but with this query, it seems that a keyword is not necessary, as any query always shows all top pathways.
- A number of nodes have ‘null’ labels e.g. if they are interaction nodes. It would be nice to be able to suppress these labels somehow.
- It would be useful to see other information associated with the nodes e.g. data source, links to external databases. Some of this information is already shown in section 6 in panel A of the import network dialog shown in Figure 1.
- Is it possible to choose another layout after a network has been loaded?
- Bug: Clicking the ‘i’ information button on the right hand button tool bar brings up a help dialog called the ‘navigation wizard’. The links to PDFs at the bottom of this screen go to non-existent web pages.
- The edge names are all ‘0’, but could be the type of edge.

 

Minor text fixes:

- “within BioLayout, the user may refine searches by keywords, species, data source and BioPAX type” -> BioPAX class type.

I have read this submission. I believe that I have an appropriate level of expertise to confirm that it is of an acceptable scientific standard.
